# Clinical Relevance of RNA Editing to Early Detection of Cancer in Human

**DOI:** 10.23937/2469-570x/1410066

**Published:** 2020-03-14

**Authors:** Mujib Ullah, Asma Akbar

**Affiliations:** Department of Radiology, School of Medicine, Stanford University, USA

## Abstract

DNA encodes RNA and is responsible for protein production in cells. RNA editing is the process by which genetic information is altered in the RNA molecule. RNA editing in cancer initiation, progression and development has been well documented and play an important role in tumorigenesis. Studying RNA editing and its application to change genetic information after transcription, RNA-editing technology could be an important innovation in cancer and has the potential for more effective precision treatment. Bioengineering integration approach and artificial intelligence could revolutionize the entire field of RNA editing for early detection of cancer.

RNA, a short-intermediate of DNA, is responsible for protein production in cells [1,2]. RNA editing is a better tool to understand how genetic information flows from DNA to protein [2,3]. In the last decade, efficient and precise RNA editing to correct disease-relevant transcripts have started to attract considerable attention for treating genetic diseases and cancer [2,4].

RNA editing involves the insertion, deletion or substitution of nucleotides within parent RNA, and alters RNA sequence without altering the sequence of genomic DNA [2,5]. Unlike DNA editing, which is permanent, the effects of RNA editing are reversible and transient [4,5]. Therefore, they would offer a new strategy for treating temporary conditions like pain or inflammation [4,5]. Many scientists are developing RNA-editing therapies, and some are trying to design new RNA editors [4]. Researchers are also designing molecules that guide our own enzymes to precisely edit RNA [4,6].

Multiple studies have recently linked RNA editing to cancer development and in metastasis of breast and many other types of cancer. Cancer is the leading cause of deaths worldwide [1,7]. One solution is to diagnose cancer at an early stage. The rapidly evolving technologies are doing much in this area but need to be expanded. Today, we propose novel RNA editing is a post-transcriptional process that alters the nucleotide sequences of certain transcripts, and can be used for diagnostic and treatment of cancer. RNA editing converting adenosines to inosines [4]. Even though RNA editing is associated with cancer development, the function and clinical relevance of editing in cancers have not been well studied [4,8].

Publicly available, DARNED and RADAR are the two main databases of RNA editing [4,9,10]. These databases can be used for bioinformatics hunting to search and identify RNA editing sites in a specific genomic location [5,9,10]. RNA editing will enable to identify early stage tumor which make it potentially been related with the process of early diagnosis in cancer [5,8]. Therefore, RNA editing enzymes such as ADARs and APOBECs are promising potential biomarkers in cancer and metastatic diseases [2,3,8,11]. RNA editing, can be used to fix the genetic mutation in cancer [3,8,11]. None of the RNA editors are perfect yet [5]. Understanding the precise role of RNA editing remains a challenge and needs further study to explore its role in cancer research.

RNA editing could be used to unravel dormant cancer stem cells that often escape chemotherapies [2,3,5]. In this way the RNA editing could be used that target therapeutic resistance and tumor relapse, and also highlights ADAR and CD9 as specific targets for cancer stem cell elimination [2,8,12,13].

We propose that engineered-guided-RNAs that bind ADAR and direct it to fix RNA mutations would bring new opportunities to identify cancer biomarkers at early stage [2,5,6]. Using RNA editing sites as a tool, many early events in the cancer progression can be identified.
